# Computational methods for cancer driver discovery: A survey

**DOI:** 10.7150/thno.52670

**Published:** 2021-03-20

**Authors:** Vu Viet Hoang Pham, Lin Liu, Cameron Bracken, Gregory Goodall, Jiuyong Li, Thuc Duy Le

**Affiliations:** 1 UniSA STEM, University of South Australia, Mawson Lakes, SA 5095, AU.; 2 Centre for Cancer Biology, SA Pathology, Adelaide, SA 5000, AU.; 3 Department of Medicine, The University of Adelaide, Adelaide, SA 5005, AU.

**Keywords:** cancer driver, cancer driver discovery, computational method, coding gene, microRNA

## Abstract

Identifying the genes responsible for driving cancer is of critical importance for directing treatment. Accordingly, multiple computational tools have been developed to facilitate this task. Due to the different methods employed by these tools, different data considered by the tools, and the rapidly evolving nature of the field, the selection of an appropriate tool for cancer driver discovery is not straightforward. This survey seeks to provide a comprehensive review of the different computational methods for discovering cancer drivers. We categorise the methods into three groups; methods for single driver identification, methods for driver module identification, and methods for identifying personalised cancer drivers. In addition to providing a “one-stop” reference of these methods, by evaluating and comparing their performance, we also provide readers the information about the different capabilities of the methods in identifying biologically significant cancer drivers. The biologically relevant information identified by these tools can be seen through the enrichment of discovered cancer drivers in GO biological processes and KEGG pathways and through our identification of a small cancer-driver cohort that is capable of stratifying patient survival.

## 1. Introduction

Identifying cancer driver genes (cancer drivers for short) is vital since these genes play a significant role in the development of cancer. Understanding cancer drivers and their regulatory mechanism is crucial to the design of effective cancer treatments.

**Figure 1:**
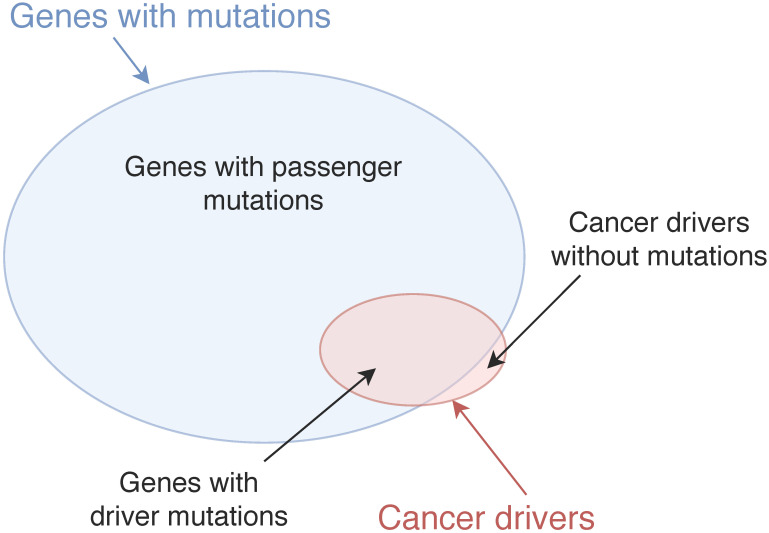
Cancer drivers and genes with mutations. Genes with driver mutations are cancer drivers. Some genes which do not contain mutations but regulate driver mutations to develop cancer are also considered as cancer drivers.

Classical methods of identifying cancer driver genes are based on detecting the mutations in the DNA sequences of coding genes in wet-lab experiments. There are many mutation types in the genome such as single-nucleotide variants (SNVs), structural variants (SVs), insertions and deletions (indels), and copy number aberrations (CNAs) 1. These mutations may cause normal cells to transform to tumour cells, resulting in the development of cancer. For example, it has been confirmed that mutations in genes *VHL* and *MET* cause kidney cancer 2 and mutations in genes *AKT1* and *BRCA1* are related to breast cancer 3. However, many mutated genes are not driver genes and may not regulate the progression of cancer. The reason is that not all mutations in the genome contribute to cancer development. Mutations which play a significant role in cancer progression are called driver mutations while mutations which do not have any impact on cancer development are called passenger mutations 4, 5. Genes which bear cancer driver mutations are considered as cancer drivers 6. Nevertheless, some cancer drivers may not contain mutations. For example, genes which may not contain mutations but regulate targets to develop cancer are also considered as cancer driver, e.g. the overexpression of KDM5C decreases p54 expression to enhance the proliferation and invasion of gastric cancer cells and *KDM5C* is considered as a cancer driver 7. The illustration of cancer drivers and genes with mutations is shown in Figure 1.

Given the complexity of the regulation by cancer drivers and the large number of genes, over twenty thousand, detecting cancer driver genes is challenging with the wet-lab experiments and many computational methods utilising multiple types of genomic data have been developed to reveal cancer drivers and their regulatory mechanism behind the cancer development 8-12. Cancer driver discovery methods are increasingly popular recently because of the fast development of machine learning techniques and significant revolution of DNA sequencing techniques. Taking these advantages, numerous methods have been proposed to detect cancer driver genes. For example, MutSigCV 13 investigates the significance of mutations in genes to predict cancer drivers, OncodriveFM 14 and OncodriveCLUST 15 evaluate the functional influence and clustering of gene mutations respectively, DriverNet 16, MEMo 17, and CBNA 18 examine the role of genes in gene regulatory networks. Due to the large number of the current computational methods for cancer driver discovery, it may take the huge amount of effort for people to find a good resource to know the state-of-the-art methods, and thus a review is necessary and helpful.

There have been previous works 1, 19 reviewing the computational methods for identifying cancer drivers. These reviews only focus on single cancer drivers (i.e. individual genes as cancer drivers) at the population level (i.e. cancer drivers for the whole population of patients of a cancer type). However, it is important to gain mechanistic insight into how cancer drivers work together in driving cancer. Besides, cancer drivers of each patient may be different from others since cancer is a heterogeneous disease, each patient has a different genome and the disease of each patient may be driven by different cancer driver genes. Thus, we also need to consider cancer driver modules and personalised cancer drivers (i.e. cancer drivers for a specific patient). In addition, there are numerous new cancer driver identification methods which have been developed since then. Therefore, it is required to have a more comprehensive review about the current computational methods for identifying cancer drivers.

In this paper, we survey computational methods for discovering both single cancer drivers and cancer driver modules at the population level and the individual level as well. We then analyse the advantages/disadvantages of the current methods and identify challenges of the field. To facilitate the development of new computational methods for cancer driver detection, we survey resources which can be used as tools in conducting cancer driver research and validating predicted cancer drivers (see **Section 2** in the Supplement). In addition, with the case study conducted to compare the performance of the current methods in this paper, we believe it will be useful for researchers, who are interested or work in the field, to develop their new methods.

The paper is structured as follows. In **Section 2**, we review computational methods for identifying single and cancer driver modules from genomic data, including cancer drivers for both the population and individuals. In **Section 3**, we carry out a case study. We analyse the current methods to identify their advantages and limitations then discuss future directions and challenges of the field in **Section 4**. Finally, we make recommendations and conclude the paper.

## 2. Cancer driver discovery methods

**Figure 2:**
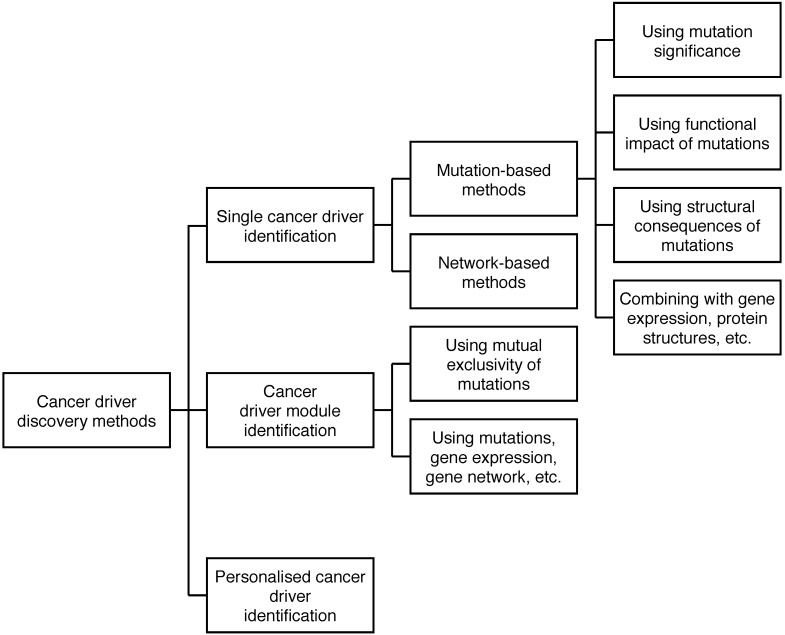
Categorisation of cancer driver discovery methods. The methods are categorised in three groups: Single cancer driver identification, Cancer driver module identification, and Personalised cancer driver identification. Single cancer driver identification includes two sub-groups: Mutation-based methods and Network-based methods. Mutation-based methods discover cancer drivers using mutation significance, functional impact of mutations, etc. Most cancer driver module identification methods use the mutual exclusivity of mutations to identify modules of cancer drivers.

The current computational methods use a wide range of genomic data types, including mutations, gene expression, pathways, etc. to discover different types of cancer drivers. Thus, we categorise the methods into various categories and sub-categories. The diagram of the categorisation is shown in Figure 2.

In the categorisation, we differentiate single cancer drivers from modules of cancer drivers. While a single cancer driver is an individual gene which initialises and progresses cancer, a module of cancer drivers is a set of genes which influence their targets to develop a certain cancer. A cancer driver module may include genes which have a mutual exclusivity of mutations 4, 17, 20, or cohesive genes which have a high density of interactions in a gene network 21. We distinguish the two types of cancer drivers since there is evidence showing that multiple genes work in concert to influence their targets in different biological processes 22, thus the roles of single genes and sets of genes in driving cancer may be different. Furthermore, as cancer is a heterogeneous disease, each patient may have a different morphology and clinical outcome. For instance, two patients, who have the same cancer type and receive the same treatment, may experience different outcomes. The reasons can come from the difference of the patients' genome or non-genomic events such as infiltration of immune cells. However, for the research of discovering possible genetic cancer drivers (which has its focus on the difference of the patients' genome), we believe it is reasonable to hypothesise that different patients' diseases could be driven by different driver genes, leading to a strong need to study cancer driver genes specific to an individual patient. Thus, we categorise the current computational methods for cancer driver discovery into three groups, including methods to identify single cancer drivers, methods to identify cancer driver modules, and methods to discover personalised cancer drivers (i.e. cancer drivers for a specific patient). In addition, based on the key techniques used in the methods, we divide single cancer driver identification methods into two sub-groups, including mutation-based methods and network-based methods.

Mutation-based methods use different characteristics of mutations such as mutation significance, functional impact of mutations, location of mutations to discover cancer drivers while network-based methods evaluate the role of genes in biological networks to predict cancer drivers. Most of cancer driver module identification methods use the mutual exclusivity of mutations to identify modules of cancer drivers. We will discuss the detail of the methods in the following sections.

### 2.1 Methods for identifying single cancer drivers

Most current methods identify single cancer drivers at the population level. In general, they can be grouped in mutation-based methods and network-based methods. Mutation-based methods use the characteristics of mutations (e.g. the significance of mutations in genes, the functional impacts of mutations, the recurrence of mutations in genes, etc.) to identify cancer driver genes while network-based methods use gene networks to assess the role of genes then combine with the mutation information to predict cancer drivers. The general idea of the network-based methods is illustrated in **Section 1** of the Supplement. The summary of the single cancer driver identification methods is presented in Table 1.

#### Methods for identifying cancer drivers based on gene mutations

Although all the mutation-based methods use mutational impact to identify cancer drivers, different methods have different hypotheses. For example, some methods (e.g. OncodriveFM, DriverML, etc.) hypothesise that mutations with functional impacts may be driver mutations while other methods (e.g. ActiveDriver, SGDriver, etc.) hypothesise that driver mutations may cluster in particular protein sections. Thus, to present the mutation-based methods in a structured way, we have grouped them by considering the mutation information used by these methods. We have divided the methods into four sub-groups, including using the significance of mutations in genes, using the functional impacts of mutations, using structural consequences of gene mutations, and others. Other methods combine the mutation information of genes with gene expression and/or tumour pathways to detect cancer drivers. The details of the methods in the four sub-groups are discussed below.

##### Using the significance of mutations in genes

MutSigCV 13 is a method to discover cancer drivers by assessing the significance of mutations in genes.

Cancer drivers predicted by MutSigCV are mutated more frequently than expected by chance based on inferred background mutation processes. However, MutSigCV has a limitation since there are still genes which have a high degree of mutations, but these mutations are passenger mutations and do not contribute to the cancer development.

##### Using the functional impacts of mutations

OncodriveFM 14 uses the functional impact of genomic mutations to detect cancer drivers instead of evaluating the significance of mutations in genes like MutSigCV. OncodriveFM hypothesises that any bias of variations (i.e. mutations) in genes with a significantly functional impact may be an indicator for identifying candidate driver genes. The significant point of this method is that instead of assessing how many mutations a gene has, it evaluates how biased mutations with highly functional impacts are. Thus, it can detect driver genes having mutations with low recurrence, but their mutations play a significant role in the cancer development.

Similar to OncodriveFM, OncodriveFML 23 also uses the functional impact of mutations to discover cancer drivers. However, while OncodriveFM only uses coding gene mutations, OncodriveFML is designed to analyse both coding and non-coding mutations. The OncodriveFML framework is then applied to 19 tumour datasets and uncovers well-known coding drivers like *TP53, KEAP1, ARID2*, and *RUNX1* with high functional impacts. It also identifies non-coding drivers such as *MALAT1* and *MIAT*. In particular, *MALAT1* is a lncRNA which has been proved to be involved in lung adenocarcinomas and *MIAT* is a non-protein-coding transcript related to myocardial infarction.

Another method assessing the functional impact of gene mutations to unravel cancer driver is DriverML 24. Different from OncodriveFM and OncodriveFML, DriverML assumes that the functional impact of mutations is affected by mutation types. Thus, it proposes a method to detect cancer drivers by scoring functional influences of alterations based on mutation types. The method uses various properties to weight the impact of mutation types and it obtains optimised weight parameters by using a supervised machine learning approach with pan-cancer training data.

##### Using structural consequences of gene mutations

Instead of using the functional impact of mutations like OncodriveFM, OncodriveFML, and DriverML, other methods, such as ActiveDriver 25, SGDriver 26, AlloDriver 27, and OncodriveCLUST 15, identify cancer drivers based on structural consequences of gene mutations. ActiveDriver discovers cancer driver genes by detecting the enrichment of somatic mutations in post-translationally modified sites, including phosphorylation, acetylation, and ubiquitination sites. SGDriver uses a Bayes inference statistical framework to incorporate somatic missense mutations into protein-ligand binding-site residues in order to figure out the functional role of the mutations. AlloDriver maps mutations to allosteric/orthosteric sites derived from the three-dimensional protein structures to detect potentially functional genes/proteins in cancer patients.

OncodriveCLUST is based on the fact that gain-of-function mutations usually cluster in particular protein sections and these mutations contribute to the development of cancer cells. Thus, it detects cancer genes with a large bias in clustering mutations. As this method bases on the mutation clustering, it cannot identify cancer drivers whose mutations are distributed across the sequence. In addition, to have a good result, it requires a large number of observed mutations. Thus, this method should be used to complement results of other methods in detecting cancer drivers.

##### Others: Combining with gene expression, pathways, protein structures, etc

The platform IntOGen-mutations 28 is developed based on OncodriveFM and OncodriveCLUST to discover cancer drivers for various tumour types. This platform uses somatic mutations, gene expression, and tumour pathways as the input parameters. It takes the advantages of both methods using the functional impact of mutations and methods using the location of mutations by applying OncodriveFM to identify driver genes which are biased significantly toward mutations with high functional impacts and applying OncodriveCLUST to detect driver genes which have mutations highly concentrating in specific regions of proteins.

Also using mutational information in detecting cancer genes, PathScan 29 combines mutations with the information of genes in known pathways. PathScan tests the scenario in which pathway mutations contribute to the development of tumour. Sakoparnig et al. 30 introduce a computational method to detect genomic alterations with low occurrence frequencies based on mutation timing.

Especially, methods such as CONEXIC 31 and ncDriver 32 combine a wide range of data types in order to identify cancer drivers more effectively. In 31, the authors develop a computational framework which uses CNVs and gene expression as the inputs to uncover cancer drivers. The framework is named COpy Number and EXpression in Cancer (CONEXIC). It applies a score-guided search to detect combinations of modulators which reflect the expression of a gene module in a set of tumour samples. Then it identifies those having the highest score in amplified or deleted regions on chromosome. The authors hypothesise that in case the expression of gene A and its copy number are related, the copy number variation likely results in changes in expression of gene A and there is a high probability that A is a driver candidate and it regulates other genes. The authors apply this framework to the dataset of melanoma and detect exactly its known cancer drivers.

In addition, ncDriver 32 identifies non-coding cancer drivers with a two-stage procedure. The first stage is mutational recurrence test which uses mutations (including indels and SNVs) and genomic elements as the inputs to detect elements with mutational recurrence. The second stage is to assess whether mutations of each element have a significant cancer-specific distribution and significant bias for highly conserved positions of each element, then it finds out if the conservation level of mutations is significantly large comparing to the overall conservation distribution. This procedure is applied to the pan-cancer whole-genome dataset to identify cancer drivers and significant non-coding drivers identified by the method are *MIR142* lncRNA and *XRNU5A-1* sncRNA.

The methods above only rely on mutations with high frequency (i.e. using mutation significance) or low frequency (i.e. combining with functional impacts of mutations, gene expression, etc.). However, according to Nussinov et al. 33-35, the mutations of driver genes can be rare mutations too. Rare mutations are different from high or low frequency mutations as they can be allosteric while high or low frequency mutations locate at active or functional sites. If mutations relieve autoinhibition, they are likely to be drivers although they are rare 34. As a result, rare drivers (i.e. drivers with rare mutations) may not be identified by these methods. Recently, there are some methods developed to identify rare cancer drivers such as HotSpot3D 36 and 3D clusters 37. HotSpot3D is a computational tool to detect three-dimensional (3D) spatial relationships in the encoded protein (i.e. spatial hotspots) and predict the protein function of mutations in the detected hotspots. Using the method, 369 rare drivers such as TP53, PTEN, VHL were detected which are all related to hotspots having potential functional implications. The 3D clusters method uses 3D protein structures to cluster somatic mutations, then it considers recurrence of mutations in clusters of spatially close residues when identifying rare drivers.

##### Analysis

Although all the methods above base on mutation data to identify cancer drivers, each has a different approach. MutSigCV evaluates the significance of mutations in genes to detect cancer drivers. However, there are still genes which are mutated significantly, but most of their mutations are passenger mutations, which do not progress cancer. Thus, these genes are not cancer driver genes. To eliminate passenger mutations, ActiveDriver, SGDriver, AlloDriver, and OncodriveCLUST consider the location of mutations. Although these methods can reduce the false positives in predicting driver mutations, they may overlook cancer drivers with mutations distributing across the protein since they only evaluate mutations which are concentrated in particular protein sections. Instead of using the location of mutations, other methods use different strategies. For instance, OncodriveFM, OncodriveFML, and DriverML utilise the functional impact of genomic mutations to evaluate the importance of mutated genes to discover cancer drivers. Sakoparnig et al. 30 bases on the timing of mutations, PathScan combines with the pathway data, and CONEXIC combines with the gene expression data. There are also methods which use an integrated approach such as IntOGen-mutations, which considers both the functional impact of mutations and their clustering as well. Furthermore, since mutations in both coding regions and non-coding regions play a significant role in cancer development, cancer drivers can be coding or non-coding elements. Methods like OncodriveFML and ncDriver are developed to detect non-coding cancer drivers.

As these methods evaluate different aspects of mutations to identify cancer drivers, they can detect several validated cancer drivers. The novel cancer drivers identified by these methods are potential and they can be used in wet-lab experiments to confirm their role in cancer progression. However, although these methods can be easily applied to different mutation datasets, mutation databases are incomplete and the applications of these methods are limited.

#### Methods for identifying cancer drivers based on gene networks

In general, network-based methods evaluate the role of genes in biological networks and then combine with the mutation information of genes to predict cancer drivers. There are three methods in this group, including Vinayagam et al. 38, CBNA 18, and DriverNet 16. The details of these methods are discussed as below.

##### The details of methods

Vinayagam et al. 38 applies controllability analysis on the directed network, i.e., the network with directed edges, of human protein-protein interaction (PPI). The input network includes nodes which are proteins and edges which are interactions between proteins. The controllability analysis categorises nodes into the three types which are ”indispensable”, “dispensable”, or ”neutral” based on their impact on minimum driver node set (MDS), i.e., the minimum node set driving the whole network. Indispensable nodes are nodes which make the number of MDS increased when the nodes are removed from the network, while dispensable nodes make the number of MDS decreased. The removal of neutral nodes from the network has no effect on the number of driver nodes. Then the study analyses the controllability of perturbated network to identify sensitive indispensable nodes, i.e., indispensable nodes in the original network but not in the perturbated network. These sensitive indispensable nodes are the candidate cancer drivers.

Also inspired by the network controllability, CBNA 18 analyses the controllability of a gene regulatory network to discover cancer drivers. However, the network built by CBNA is a miRNA-TF-mRNA network which consists of microRNAs (miRNAs), Transcription Factors (TFs), and mRNAs. Since this network is constructed from the expression data of miRNAs/mRNAs of cancer patients and the existing gene interaction databases such as PPI 39, miRTarBase 40, and TransmiR 41, it is more reliable and specific to a cancer type. In addition, different from the method of Vinayagam et al. 38, CBNA analyses the network controllability to indicate critical nodes of the network, i.e. nodes increase the number of the minimum node set controlling the whole network if they are removed from the network, then combining with the mutation data to identify cancer drivers. As CBNA uses the miRNA-TF-mRNA network, it can identify both coding and miRNA driver genes. Furthermore, it can also be used to discover drivers for a cancer type or cancer subtype.

Instead of evaluating the controllability of a subset of nodes of a gene network like Vinayagam et al. 38 and CBNA 18, DriverNet 16 considers the influence of mutated genes on other genes in a network.

DriverNet integrates different data types, including genome data (i.e. non-synonym SVNs, indels, and copy number variation), influence graph of biological pathway information, and gene expression. It constructs a bipartite graph of genes to detect the effect of mutated genes on genes which have an outlying expression. The putative drivers are mutated genes which impact on a high number of outlying-expression genes in several patients. The method is applied to four cancer datasets, including glioblastoma, breast, triple negative breast, and serous ovarian, and it reveals various candidate cancer drivers related to transcriptional networks.

##### Analysis

The three methods above use biological networks to predict single cancer drivers, other methods using networks to discover cancer driver modules or personalised cancer drivers are discussed in **Section 2.2 and 2.3** respectively. In general, network-based methods evaluate the role of genes in the whole networks to predict cancer drivers. Various techniques are used to analyse the networks such as network controllability in Vinayagam et al. and CBNA or the influence of genes in DriverNet. These methods can elucidate molecular mechanisms in cancer development at the network level, but they need large datasets to produce reliable results. In addition, the networks used in some methods (i.e. Vinayagam et al. and DriverNet) are not specific to any cancer type, thus they may miss the important information which is specific to a cancer type. Another limitation of network-based methods like DriverNet is predicting genes which affect other genes' expression as cancer drivers, because some cancer drivers may not alter the expression of other genes or other genes accidentally change other genes' expression although they are not cancer drivers.

### 2.2 Methods for identifying cancer driver modules

Recently, several methods have been developed to discover cancer drivers in modules. Most of the methods identifying cancer driver modules use mutual exclusivity of mutations. Thus, we divide methods for identifying cancer driver modules into two sub-groups: using mutual exclusivity of mutations and others. Other methods use mutations, gene expression, gene network, RNA sequencing, etc. to detect cancer driver modules. The details of methods in the two sub-groups are discussed as below and the summary of the methods is presented in Table 2.

#### Using mutual exclusivity of mutations

CoMEt (the Combinations of Mutually Exclusive Alterations) 4 uses mutual exclusivity technique to detect cancer driver modules. Because different cancer patients have different combinations of genomic alterations which develop the disease, CoMEt detects combinations of alterations (i.e. modules of mutated genes) in the same pathway, which are mutual exclusive across samples. The method uses the exact statistical test to test mutual exclusivity and it does simultaneous analysis for mutually exclusive alterations specific to cancer subtypes. The advantage of this method is that it has a low computational complexity. Similarly, WeSME 20 also assesses the mutual exclusivity of mutations of genes to detect cancer drivers. However, instead of evaluating genes in the same pathway, WeSME only considers gene pairs and the gene pairs whose mutations have a significantly mutual exclusivity are considered as modular candidate cancer drivers.

MEMo (Mutual Exclusivity Modules) 17 applies mutual exclusivity technique in biological networks to identify oncogenic network modules. According to 17, although individual tumours of the same cancer type may have different genomic alterations, these alterations just happen in a restricted number of pathways. In addition, alterations in the same pathway are not likely to exist in the same patient. Based on these, MEMo does correlation analysis and applies statistical tests to detect network modules based on three criteria: (1) genes in a network module are altered across the sample; (2) member genes tend to join into the same biological process; (3) alterations in modules are mutually exclusive. The method is applied to the glioblastoma multiforme (GBM) dataset and detects successfully known network modules, i.e., groups of cancer drivers, in GBM.

#### Others: Using mutations, gene expression, gene network, RNA sequencing, etc

iMCMC (an approach to identify Mutated Core Modules in Cancer) 42 is developed to uncover groups of genes driving cancer using the cancer genomic data from cancer patients. The method uses somatic mutation, CNV, and gene expression to build a gene network. Then, it identifies coherent subnetworks (modules) from the network through an optimisation model by selecting vertices and edges with high weights. Finally, the significance of subnetworks is assessed by performing a random test and the mutual exclusivity of subnetworks is tested by adopting Markov chain Monte Carlo permutation strategy. The method is applied to the GBM and the ovarian carcinoma (OV) datasets from TCGA. Many discovered core modules are related to known pathways and most of the identified genes are cancer driver genes which are already reported relating to cancer pathogenesis in other research.

NetBox 21 uses biological networks in studying drivers for GBM. It introduces a network-based method to detect oncogenic processes and cancer driver genes. The hypothesis of the approach is that biological networks include multiple functional modules, and tumours target specific functional modules. The method analyses sequence mutations, CNVs, an interaction network including both PPIs and signalling pathways to identify and assess network modules statistically.

Another method to identify cancer driver modules is TieDIE (Tied Diffusion through Interacting Events) 43. TieDIE applies network diffusion to discover the relationship of genomic events and changes in cancer subtypes. The approach collects a subnetwork of PPIs, interactions of genomic perturbations, predicted transcription factor-to-target connections, and transcriptomic states from literature. The method is applied to the breast adenocarcinoma (BRCA) dataset of TCGA and it detects signalling pathways and interlinking genes corresponding to cancer signalling.

CICERO 44 has a different approach in identifying cancer driver modules. It considers gene fusions, the results from genomic structural variations, as drivers which can initialise and develop cancer. Thus, it uses RNA sequencing data and extensive annotation to detect driver fusions with a local assembly-based algorithm.

The methods above identify coding cancer driver modules. However, because non-coding RNAs (e.g. miRNAs) can modulate tumorigenesis by promoting or suppressing specific genes and various cancer types have overlaps in oncogenic pathways, a group of miRNAs which drives or suppresses tumorigenesis in different tumour types may exist. Hamilton et al. 45 use the pan-cancer dataset of TCGA and the miRNA target data of Argonaute Crosslinking Immunoprecipitation (AGO-CLIP) 46-48 to detect pan-cancer miRNA drivers. The idea is that the set of cancer miRNA drivers will modulate tumorigenesis and share a central core seed motif. The result shows that an oncogenic miRNA superfamily, which includes *miR-17, miR-18, miR-19, miR-93, miR-130, miR-210*, and *miR-455*, coregulates tumour suppressors through a *GUGC* core motif.

#### Analysis

As can be seen from the methods above, most of the methods use mutual exclusivity of mutations to identify cancer driver modules. With this technique, the mutation from only one member in an identified module is enough to trigger cancer progression 20, 49. Thus, the identified drivers in a module may not work together to regulate their targets to drive cancer. However, as discussed above, genes should collaborate to increase their influence on target genes to progress cancer. Therefore, it is necessary to develop novel methods to discover cancer driver groups whose members work in concert to initialise and develop cancer.

### 2.3 Methods for identifying personalised cancer drivers

The methods discussed in **Section 2.1 and 2.2** discover cancer drivers at the population level. Since different patients possess different genomes and their diseases might be driven by different driver genes 50, it is necessary to investigate cancer drivers which are specific to an individual patient (i.e. personalised cancer drivers). There are three methods in this group, including DawnRank 51, SCS 52, and PNC 53. All of them base on gene regulatory networks to predict personalised cancer drivers. The details of these methods are discussed as below, and the summary of the methods is presented in Table 3.

#### The details of methods

A representative of methods for identifying personalised cancer drivers is DawnRank 51. In general, the idea of the method is that mutations in genes which have higher connectivity in an interaction network are more impactful. DawnRank uses the information of gene expression and gene network as the inputs. In particular, it is a ranking framework which applies PageRank 54, 55 to evaluate the impact of genes on the gene network. The impact is presented in terms of network connectivity and the number of downstream genes expressed differentially. The higher the rank of a gene is, the more downstream genes it has effects on in the gene network. Ranks of genes are then combined with somatic alteration data like copy number variations to detect driver alterations. Although DawnRank bases on the same gene regulatory network for all patients, it assesses the impact of genes in each patient using the patient's gene expression data to detect personalised cancer drivers. The algorithm has been applied to TCGA datasets and it shows effectiveness in detecting cancer drivers.

To assess the impact of genes in each patient, DawnRank uses the gene expression data of each patient, but it bases on the same gene regulatory network of all patients. As a result, it may miss important information of gene regulation of each patient. Thus, to detect personalised cancer drivers, SCS 52 builds a gene regulatory network for each patient from the patient's gene expression data and its neighbour's gene expression data (i.e. the corresponding normal sample's gene expression data). SCS detects cancer driver genes as the minimal set of mutated genes which impacts on the maximal differentially expressed genes. Like SCS, PNC 53 also uses the gene expression data of a patient and its neighbour to construct personalised networks. Nevertheless, PNC only selects edges which are different between the tumour and normal state. It then converts the gene regulatory network to a bipartite graph in which, nodes on the top represent genes and nodes on the bottom represent edges. PNC predicts cancer driver genes as the minimum gene set on the top of the bipartite graph which covers all the edges on the bottom.

#### Analysis

Although these methods can discover personalised cancer drivers, they still have some limitations.

DawnRank bases on the same gene network of all patients. It ignores the network information specific to an individual patient, leading to false positives in its results. On the other hand, SCS and PNC use the genetic data of each patient to construct personalised gene networks. However, they require the genetic data of a pair of samples (i.e. a tumour and its tumour neighbour) but identifying the neighbour of a tumour is challenging and it is not always existing. In addition, these methods only discover coding cancer drivers while non-coding genes (e.g. miRNAs) can also be cancer drivers as discussed above.

## 3. A comparative study of cancer driver discovery methods

### 3.1 Performance of methods in identifying cancer drivers

In this section, we present a comparative study to compare the performance of the methods above. As there is not a ground truth to compare the results of methods for discovering cancer driver modules, we only select five methods for identifying single cancer drivers and three methods for identifying personalised cancer drivers for the comparison, including ActiveDriver 25, DawnRank 51, DriverML 24, DriverNet 16, MutSigCV 13, OncodriveFM 14, PNC 53, and SCS 52. These methods represent for different approaches in detecting cancer driver genes. ActiveDriver, DriverML, MutSigCV, and OncodriveFM are mutation-based methods while DawnRank, DriverNet, PNC, and SCS are network-based methods. In addition, DawnRank, PNC, and SCS identify personalised cancer drivers while other five methods identify cancer drivers at the population level. Although DawnRank, PNC, and SCS detect cancer drivers for each patient, they all have a method to aggregate the results of individual patients to predict cancer drivers for the population. Thus, we can compare these three methods with the others. The comparison is performed based on the results of the eight methods in identifying drivers for breast invasive carcinoma (BRCA), lung adenocarcinoma (LUAD), lung squamous cell carcinoma (LUSC), kidney renal clear cell carcinoma (KIRC), head and neck squamous cell carcinoma (HNSC). We obtain the predicted cancer drivers of the eight methods for the selected five cancer types from 53.

Since there is not a real ground truth for cancer driver discovery (and many other biological researches), it is a common practice to evaluate the findings by computational methods against the information in high quality databases such as the CGC of COSMIC, although the CGC only includes driver genes which are manually curated or predicted by multiple methods. In this comparative study, we also use the CGC as the ground truth to validate the cancer drivers predicted by the methods. The performance of a method is measured using *F_1_Score* based on the number of discovered cancer drivers that are validated by the CGC. The *F_1_Score* indicates the enrichment ability of discovered cancer drivers in the gold standard (i.e. the CGC) and it is computed based on Precision *P* and Recall *R* as shown in Eq. 1. The higher the *F_1_Score* a method has, the better the method is.


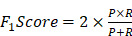
(1)

In Eq. 1, P (Precision) shows the fraction of predicted driver genes in the CGC among the predicted driver genes and R (Recall) indicates the fraction of predicted driver genes in the CGC among the driver genes in the CGC. As *F_1_Score* is computed from Precision *P* and Recall *R*, it will indicate both the ability to predict exactly cancer drivers and the ability to predict many confirmed cancer drivers of a method.

**Figure 3:**
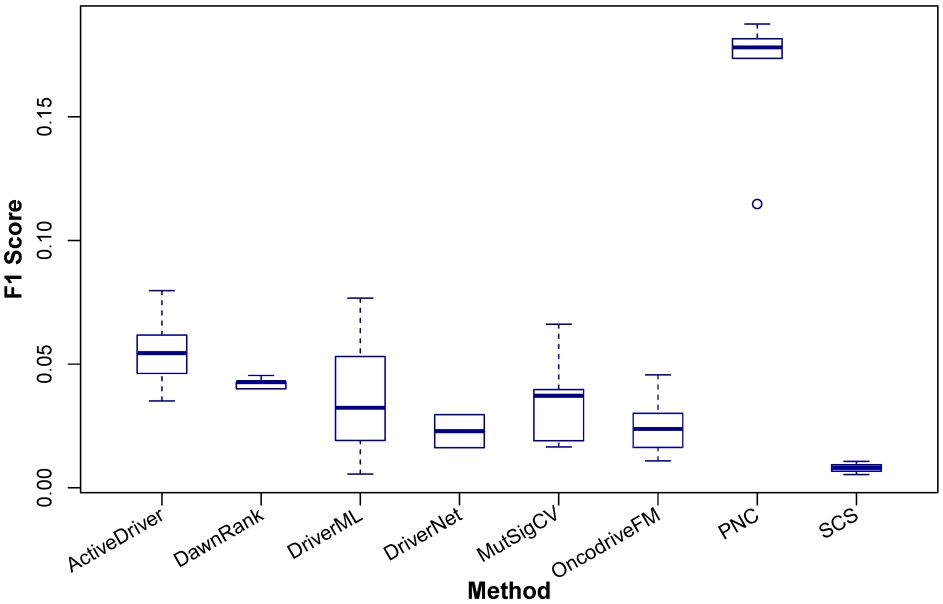
Comparison of *F_1_Score* of ActiveDriver, DawnRank, DriverML, DriverNet, MutSigCV, OncodriveFM, PNC, and SCS in identifying coding cancer drivers at the population level. The x-axis indicates the eight methods and the y-axis shows the *F_1_Score*. The results are based on the cancer driver prediction for the five cancer types, including BRCA, LUAD, LUSC, KIRC, and HNSC, of the eight methods.

The comparison result is shown in Figure 3 and the details are shown in Table 4. It can be seen that with the four data sets of BRCA, LUAD, LUSC, and KIRC samples, PNC outperforms the other methods and with HNSC, ActiveDriver has the best performance.

Given the complexity of cancer, it is not practical to include all cancer types in the comparison, so based upon the availability of data, we have chosen the five data sets such that a sufficiently large comparison between the available tools can be done to identify differences in the performance of the tools, not just between each other but also between cancer types. For example, ActiveDriver outperforms the others in HNSC, whilst PNC is superior in the other four cancer types. A method used for different cancer types is likely to yield different results so regardless of the number of cancer types selected here, it remains incumbent for the user to run their required analyses.

**Figure 4:**
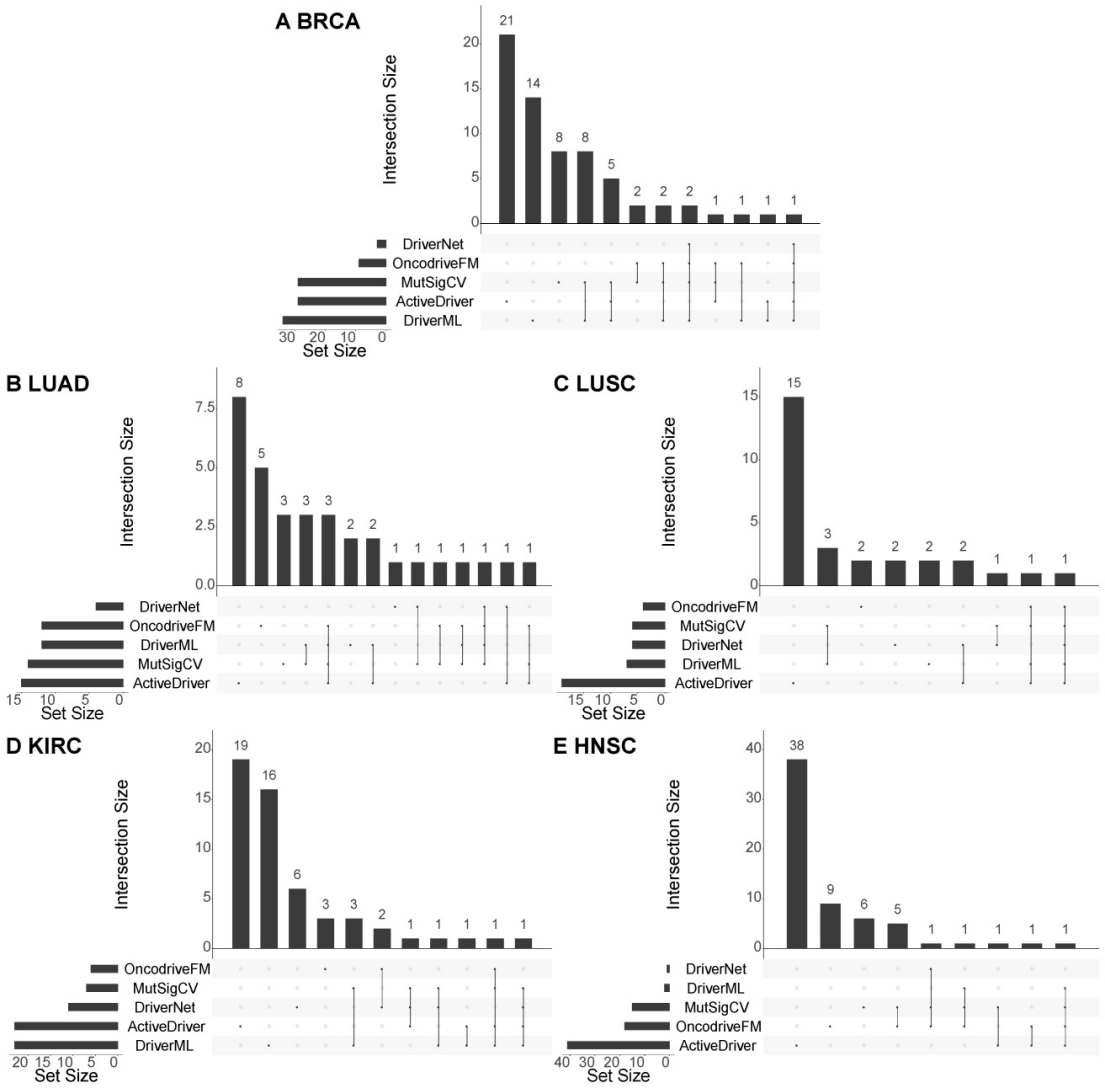
Overlap among the cancer drivers predicted by different methods. The charts illustrate the overlap among the cancer drivers at the population level predicted by the five methods (DriverML, ActiveDriver, DriverNet, MutSigCV, and OncodriveFM) w.r.t the five cancer types, including BRCA, LUAD, LUSC, KIRC, and HNSC. In each chart, the horizontal bars at the bottom left show the number of detected cancer drivers validated by the CGC, the vertical bars and the dotted lines show the overlap of the validated cancer drivers of the methods. If there is not an overlap, it will be a black dot.

Moreover, to see if the methods detect similar cancer drivers, we compare the results of the five methods used for identifying cancer drivers at the population level (i.e. DriverML, ActiveDriver, DriverNet, MutSigCV, and OncodriveFM). Figure 4 shows the overlap between the validated cancer drivers discovered by each pair of the methods, for each of the five cancer types. It can be seen that there is little overlap among the results of the methods. For example, in breast cancer, only one cancer driver (*TP53*) is identified by all the five methods, two cancer drivers (*CDH1* and *PIK3CA*) are detected by four methods (DriverML, DriverNet, MutSigCV, and OncodriveFM), and eight cancer drivers (*GATA3, NCOR1, PTEN, ARID1A, FOXA1, PIK3R1, CTCF,* and *ERBB2*) are predicted by three methods (see the detailed overlap of the predicted driver genes in **Section 3** of the Supplement). As the results of these methods are complementary, they should be used together to maximize the overall performance of the cancer driver prediction. In addition, it should be pointed out that although the CGC is popular in validating cancer drivers in cancer research, it is incomplete in the sense that the database is constantly being updated when new cancer drivers come to light. Therefore, although some of the predicted cancer drivers cannot be validated with existing knowledge, they can be novel cancer drivers which is worth for wet-lab experiments to confirm their roles in progressing cancer.

### 3.2 Identified cancer drivers enriched significantly in GO biological processes and KEGG pathways

To have a detailed look at the discovered cancer drivers, we take breast cancer as an example for the further analysis. Breast cancer is selected as the breast cancer dataset has the largest number of samples among all the available cancer datasets. We combine all the breast cancer drivers predicted by the five methods (DriverML, ActiveDriver, DriverNet, MutSigCV, and OncodriveFM) at the population level, which results in altogether 509 cancer drivers. Among them, 63 drivers are predicted by at least two of the five methods (see the details of these 63 driver genes in **Section 3** of the Supplement). We use Enrichr 56 to do enrichment analysis of these 63 drivers. Table 5 and Table 6 show the GO biological processes and KEGG pathways in which these cancer drivers are significantly enriched (adjusted p-value less than 0.05). Among the 63 driver genes, 16 genes (25.4%) are enriched in 7 GO biological processes and 15 genes (23.8%) are enriched in 26 KEGG pathways related to breast cancer. It indicates that the predicted cancer drivers are closely associated with the biological condition of breast cancer and biologically meaningful.

### 3.3 Identified cancer drivers are useful in predicting survival

**Figure 5:**
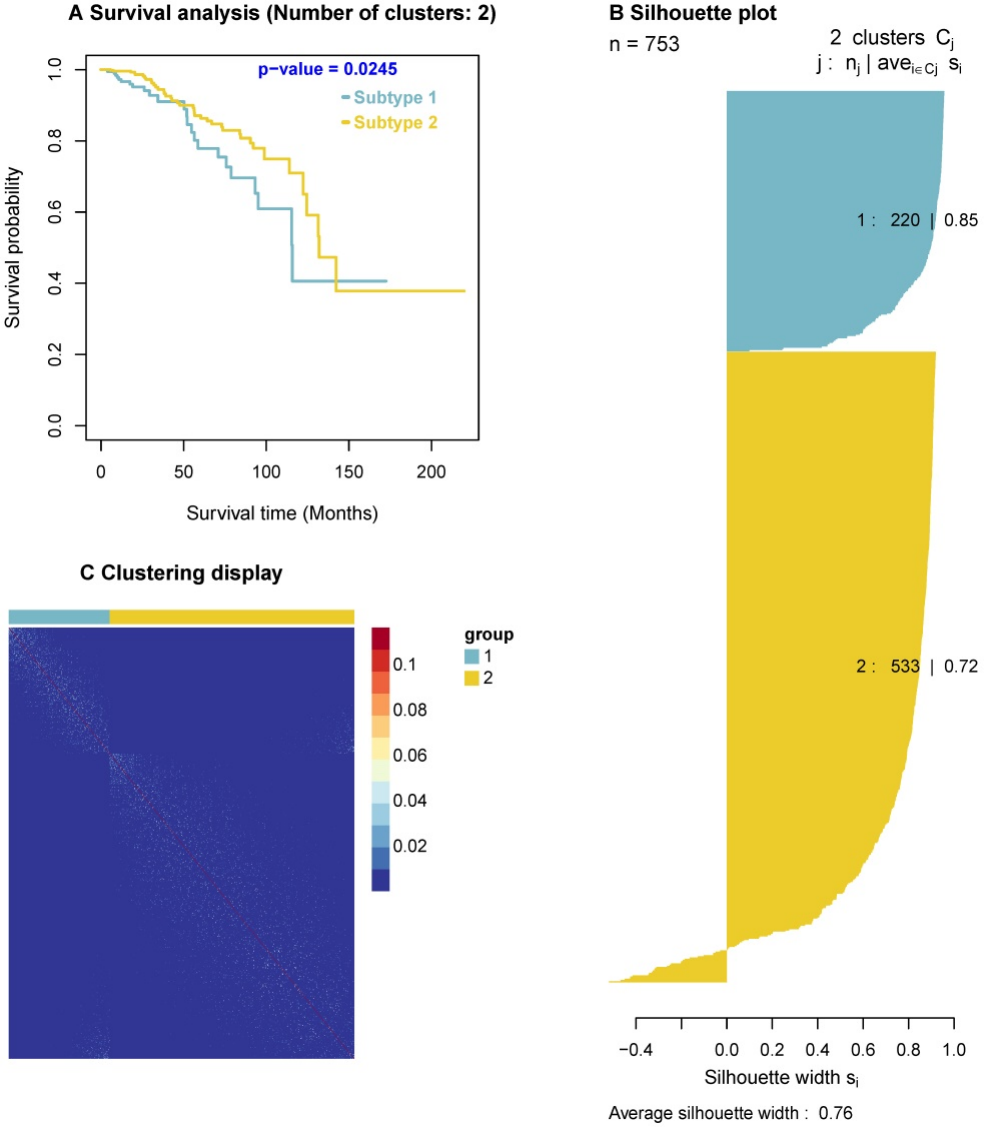
Survival curves, clustering display, and silhouette plot. Survival curves are for cancer subtypes identified by using the four predicted cancer drivers, including *AKT1, PTEN, CDKN1B,* and *TP53*. The survival curves show the significant difference in the survivals of patients of the two subtypes (p-value = 0.0245). The clustering display indicates a highly qualified clustering with the similarity of samples in each subtype (i.e. Light dots show the similarity of samples). The silhouette plot has a large average silhouette width (0.76/1), indicating the clustering validity when using these four genes.

Since the predicted cancer driver genes likely cause carcinogenesis, they could be used as biomarkers to classify tumours. To explore this concept, we use the predicted drivers to stratify breast cancer patients. Among the 63 predicted cancer drivers above, there are four significant genes, *AKT1, PTEN, CDKN1B,* and *TP53*, which are enriched in both GO biological processes and KEGG pathways. For instance, *AKT1* are enriched in two GO biological processes and 25 KEGG pathways, *PTEN* are enriched in two GO biological processes and five KEGG pathways. Thus, we use these four genes for this analysis. In addition, we obtain the BRCA gene expression data and clinical data from 57, and use the Similarity Network Fusion (SNF) method 58, 59, a popular method for discovering the similarities among patients, to cluster cancer patients. The SNF takes expression of these four genes as input and outputs subtypes of cancer patients. We then analyse the survival outcomes of patients in the classified subtypes. The results indicate that the survival level of patients in different classified subtypes are significantly different (p-value = 0.0245) as shown in Figure 5. Furthermore, the clustering display shows the similarity of samples in each identified subtype and the silhouette plot indicates a good clustering with a large average silhouette width (0.76).

## 4. Gaps and future directions

From the discussion above, we see that there are a wide range of computational methods for identifying cancer drivers from genomic data. In this paper, we categorise the methods into three groups: methods for identifying single cancer drivers (including mutation-based methods and network-based methods), methods for identifying cancer driver modules, and methods for identifying personalised cancer drivers. Although these methods have detected successfully various cancer drivers, there are still several gaps in the research of the field.

Firstly, most of the current methods focus on coding mutations to identify coding cancer drivers while non-coding cancer drivers are not fully examined and the number of methods for identifying non-coding drivers is limited. However, non-coding cancer drivers are important because protein-coding regions account for only around two percent of the human genome. The large part of mutations exist in non-coding regions and these mutations can regulate the expression of genes and drive cancer 60, 61. In addition to the limited number of non-coding cancer driver identification methods, the current methods focus much on non-coding mutations, i.e., correlations of mutations in non-coding elements with other factors like survival 32. Nevertheless, cancer drivers can be non-coding RNAs without mutations, but they can regulate other genes to progress cancer, thus it is required to investigate non-coding RNAs with and without mutations to detect non-coding cancer drivers.

Secondly, some methods have been developed to identify groups of cancer drivers 17, 42, but they are mostly based on mutations to detect mutated modules, called cancer driver modules. Since in a module, the mutation of a member is sufficient to develop cancer, the identified drivers in a module may not in fact work together to regulate their targets to drive cancer. However, there is evidence that some genes work in concert to regulate other genes' expression and influence different biological processes, such as the cooperation of miRNAs in EMT, the transformation of epithelial cells into mesenchymal cells 22, 62. In addition, in some biological processes, the regulation of single genes might not have significant impacts and research has emerged to use wet-lab experiments to investigate the regulatory of group-based regulators in biological processes. All of these highlight the importance of studying biological factors in groups, and computational methods which utilise a variety of data and techniques are in demand for investigating groups of drivers.

Finally, although there have been methods for detecting personalised cancer drivers 51-53, they still have some limitations. Some methods, such as DawnRank, use the gene network of the population to predict personalised cancer drivers. This leads to that they may ignore the information of the gene network specific to an individual patient and they may discover many false positives in their results. Other methods, such as SCS and PNC, use the personal genetic data to build personalised gene networks but they need the genetic data of a sample pair (i.e. a cancer patient and its neighbour in the normal state). The neighbour of a cancer patient is not always existing. Thus, the application of these methods is limited. Furthermore, these methods only detect coding cancer drivers while it is also necessary to identify non-coding cancer drivers as the discussion above. All of these indicate that there is a strong need to develop novel computational methods for detecting personalised coding/non-coding cancer drivers.

## 5. Recommendation and conclusion

We have investigated a wide range of computational methods for identifying cancer drivers from genomic data. In addition, the advantages and limitations of the surveyed methods are analysed, based on which we identify various opportunities for the development of the research in the field. It is clear that the research in computational approach to cancer driver identification is still in its growth phase. Much more work needs to be done and many opportunities exist in this area. Nevertheless, there are also different challenges in advancing the research in cancer driver identification. Identifying exactly biological factors which drive cancer is quite complicated. Future research needs to focus on both coding and non-coding datasets to identify candidate cancer drivers. To improve the accuracy of the novel computational methods, we should combine different types of data such as gene expression, mutations, and clinical information, etc. to detect cancer drivers.

We have also surveyed available resources which can be used in the research of discovering cancer drivers. The existing resources are plentiful, but they are fragmented. Thus, to utilise cancer data more effectively for the research, it requires to have policies to achieve better data sharing. In addition, another difficulty when developing computational methods for uncovering non-coding cancer drivers is the validation. The reason is that most of the current databases are for coding cancer drivers and there is no one for non-coding cancer drivers. Therefore, we make an urgent call for the building of databases for non-coding drivers given their crucial role in the success of the research in the field.

To evaluate the performance of the current methods in detecting cancer drivers as well as provide an example of the evaluation of cancer driver discovery methods for the researchers who would like to penetrate the field, a comparative study has been conducted. From the results of the experiment in the comparative study, it can be seen that each method can uncover different cancer drivers and the overlaps between the results of the methods are small. Therefore, the methods are complementary, and we should use them together to maximize the effectiveness of cancer driver prediction of the methods. This is also an indicator for the different approaches of the methods and to achieve a significant result, novel methods should combine various resources and techniques in detecting cancer drivers.

In conclusion, although computational methods may never completely replace wet laboratory experiments to validate biological findings, it is widely acknowledged that the predicted drivers by computational methods can be used as candidates for further wet laboratory experiments to confirm their roles in cancer development. While there are numerous computational methods for discovering cancer drivers now, there exist various gaps and opportunities for advancing the research of the field. However, due to the complexity of cancer initialisation and development, identifying cancer drivers faces many challenges. Through this paper, we hope that we can help researchers who are interested in the field to establish a solid background and motivate them to tackle the current challenges.

## Supplementary Material

Supplementary figures and tables.

## Figures and Tables

**Table 1:** Summary of methods for identifying single cancer drivers

Method	Description and reference	Additional information
***Single cancer driver identification***		
**Mutation-based methods (using mutation significance)**		
MutSigCV	Assesses the significance of mutations in DNA sequencing to discover cancer driver genes 13	The result includes false positives (i.e. passenger mutations with a high degree)
**Mutation-based methods (using functional impact of mutations)**		
OncodriveFM	Uses the functional impact of mutations of genes to detect cancer drivers with the hypothesis that any bias of variations with a significantly functional impact in genes can be used to identify candidate driver genes 14	It can identify driver genes with low mutation recurrence
OncodriveFML	Uses the functional impact of gene mutations to reveal both coding and non-coding drivers 23	It is applied to 19 cancer datasets and detects several well-known drivers
DriverML	Uses the functional impact of mutations to unravel cancer drivers through a supervised machine learning approach 24	It can be improved if integrating additional well-annotated datasets (e.g. CGC) into the training data
**Mutation-based methods (using structural consequences of gene mutations)**		
ActiveDriver	Looks at the enrichment of mutations in externally defined regions to uncover cancer driver genes 25	It only analyses missense mutations while other mutations are also important such as in frame del, frame shift del, etc.
SGDriver	Uses a Bayes inference statistical framework to incorporate somatic missense mutations into protein-ligand binding-site residues in order to figure out the functional role of the mutations 26	It can be improved if integrating more mutation types and using molecular network to identify the interacting partners of mutated proteins to expand the candidate pool
AlloDriver	Maps mutations to allosteric/orthosteric sites derived from the three-dimensional protein structures to detect potentially functional genes/proteins in cancer patients 27	It also uses only missense mutations
OncodriveCLUST	Detects cancer genes with a large bias in clustering mutations based on the idea that gain-of-function mutations usually cluster in particular protein sections and these mutations contribute to the development of cancer cells 15	It cannot identify cancer drivers whose mutations are distributed across the sequence
**Mutation-based methods (others: combining with gene expression, pathways, protein structures)**		
IntOGen-mutations	Uses somatic mutations, gene expression, and tumour pathways to identify cancer drivers for various tumour types by combining OncodriveFM and OncodriveCLUST 28	It can discover driver mutations which are distributed across the sequence and have significant functional impacts
PathScan	Combines genomic mutations with the information of genes in known pathways to uncover cancer driver genes 29	It can be extended to integrate other types of genetic anomalies
Sakoparnig et al.	Introduces a computational method to detect genomic alterations with low occurrence frequencies based on mutation timing 30	It may not discover drivers which are already present at very early cancer stages as we cannot observe a steep rise for them
CONEXIC	Applies a score-guided search to detect combinations of modulators which reflect the expression of a gene module in a set of tumour samples then it identifies those which have the highest score in amplified or deleted regions 31	It is mainly bases on copy number aberrations
ncDriver	Screens non-coding mutations with conservations and cancer specificity to reveal non-coding cancer drivers 32	It tests both recurrence and distribution of mutations to identify cancer drivers
HotSpot3D	Identifies spatial hotspots to interpret the function of mutations in the encoded protein 36	It can detect rare cancer drivers
3D clusters	Clusters somatic mutations in cancer to identify rare mutations based on 3D protein structures 37	It is limited due to the lack of complete protein structure data for several genes
**Network-based methods**		
Vinayagam et al.	Applies controllability analysis on the directed network of human protein-protein interaction to identify disease genes 38	As it uses a general protein network (i.e. not specific for a cancer type), uncovered drivers are not particular for any cancer type
CBNA	Identifies coding and miRNA cancer drivers by analysing the controllability of the miRNA-TF-mRNA network and mutation data 18	It builds the gene network for a specific cancer type, thus the results are for the cancer type of interest
DriverNet	Uncovers cancer drivers by evaluating the influence of mutations on transcriptional networks in cancer 16	It relies on a predetermined influence graph which is sparse and incomplete

**Table 2:** Summary of methods for identifying cancer driver modules

Method	Description and reference	Additional information
***Cancer driver module identification***		
**Using mutual exclusivity of mutations**		
CoMEt	Identifies cancer genes by using the exact statistical test to test mutual exclusivity of genomic events and applies techniques to do simultaneous analysis for mutually exclusive alterations 4	It has a low computational complexity
WeSME	Discovers cancer drivers by evaluating the mutual exclusivity of mutations of gene pairs 20	It can only detect driver gene pairs (i.e. only two driver genes in each module)
MEMo	Analyses mutual exclusivity of mutated genes in subnetworks to identify mutual exclusivity modules in cancer 17	It depends on the prior biological knowledge of gene interactions
**Others: using mutations, gene expression, gene network**		
iMCMC	Uses the cancer genomic data including mutations, CNAs, and gene expression from cancer patients to identify mutated core modules in cancer 42	It provides flexibility by using two input parameters to balance different sources of data
NetBox	Uses biological networks to assess network modules statistically and identify core pathways in GBM 21	It is only used for Glioblastoma
TieDIE	Applies network diffusion to discover the relationship of genomic events and changes in cancer subtypes 43	It has a high computational cost
CICERO	Uses RNA sequencing data and extensive annotation to detect driver fusions with a local assembly-based algorithm 44	It may miss low-expressed gene fusions
Hamilton et al.	Uses the pan-cancer dataset of TCGA and the miRNA target data of AGO-CLIP to detect a pan-cancer oncogenic miRNA superfamily with a central core seed motif 45	It discovers a miRNA driver superfamily consisting of *miR-17, miR-19, miR-130, miR- 93, miR-18, miR-455* and *miR-210*

**Table 3:** Summary of methods for identifying personalised cancer drivers

Method	Description and reference	Additional information
**Personalised cancer driver identification**		
DawnRank	A ranking framework which applies PageRank to evaluate the impact of genes in an interaction network to detect cancer drivers 51	It bases on the same gene network for all patients, thus may reduce the personalised information
SCS	Detects the minimal set of mutated genes controlling the maximal differentially expressed genes as cancer drivers 52	It builds a gene network for each patient; its application is limited as it requires the corresponding normal sample for each patient
PNC	Identifies cancer drivers as the minimum gene set which covers all the edges based on a bipartite graph 53	It also requires the corresponding normal sample for each patient

**Table 4:** *F_1_Score* of the eight methods in predicting drivers for the five cancer types

No.	Method	BRCA	LUAD	LUSC	KIRC	HNSC
1	ActiveDriver	0.062	0.035	0.046	0.054	0.080
2	DawnRank	0.045	0.043	0.040	0.040	0.043
3	DriverML	0.077	0.032	0.019	0.053	0.006
4	DriverNet	NA	NA	0.016	0.030	NA
5	MutSigCV	0.066	0.037	0.016	0.019	0.040
6	OncodriveFM	0.024	0.030	0.0101	0.016	0.046
7	PNC	0.178	0.174	0.182	0.188	0.115
8	SCS	NA	0.011	0.005	0.008	NA

**Table 5:** GO biological processes involved in breast cancer in which the predicted cancer drivers are enriched

Term	#Genes	p-value
GO:0045598 regulation of fat cell differentiation	5	2.0e-03
GO:0045596 negative regulation of cell differentiation	6	3.6e-03
GO:0045604 regulation of epidermal cell differentiation	3	1.2e-02
GO:0042127 regulation of cell proliferation	10	2.5e-02
GO:0045599 negative regulation of fat cell differentiation	3	2.8e-02
GO:0045580 regulation of T cell differentiation	3	2.9e-02
GO:2000736 regulation of stem cell differentiation	4	3.1e-02

**Table 6:** KEGG pathways involved in breast cancer in which the predicted cancer drivers are enriched

Term	#Genes	p-value
ErbB signaling pathway	6	5.3e-06
Thyroid hormone signaling pathway	6	2.8e-05
Sphingolipid signaling pathway	6	3.1e-05
Neurotrophin signaling pathway	6	3.0e-05
PI3K-Akt signaling pathway	8	1.7e-04
AGE-RAGE signaling pathway in diabetic complications	5	1.7e-04
HIF-1 signaling pathway	5	1.7e-04
FoxO signaling pathway	5	5.1e-04
Fc epsilon RI signaling pathway	4	5.2e-04
Toll-like receptor signaling pathway	4	2.2e-03
TNF signaling pathway	4	2.7e-03
Relaxin signaling pathway	4	4.6e-03
VEGF signaling pathway	3	5.1e-03
Estrogen signaling pathway	4	5.3e-03
mTOR signaling pathway	4	7.3e-03
Prolactin signaling pathway	3	7.4e-03
B cell receptor signaling pathway	3	7.6e-03
p53 signaling pathway	3	7.8e-03
MAPK signaling pathway	5	1.2e-02
T cell receptor signaling pathway	3	1.8e-02
Rap1 signaling pathway	4	1.8e-02
C-type lectin receptor signaling pathway	3	1.9e-02
AMPK signaling pathway	3	2.6e-02
Apelin signaling pathway	3	3.5e-02
Insulin signaling pathway	3	3.4e-02
Phospholipase D signaling pathway	3	4.1e-02

## Abbreviations

AGCOH: Atlas of Genetics and Cytogenetics in Oncology and Haematology
AGO-CLIP: Argonaute Crosslinking Immunoprecipitation
BRCA: breast adenocarcinoma
CCLE: Cancer Cell Line Encyclopedia
CNA: copy number aberration
CoMEt: Combinations of Mutually Exclusive Alterations
CONEXIC: COpy Number and EXpression In Cancer
DGIdb: Drug-Gene Interaction database
GBM: glioblastoma multiforme
GDC: Genomic Data Commons
HNSC: head and neck squamous cell carcinoma
iMCMC: identify Mutated Core Modules in Cancer
indels: insertions and deletions
KIRC: kidney renal clear cell carcinoma
LUAD: lung adenocarcinoma
LUSC: lung squamous cell carcinoma
MEMo: Mutual Exclusivity Modules
MDS: minimum driver node set
miRNA: microRNA
NCG: Network of Cancer Genes
OV: ovarian carcinoma
PPI: protein-protein interaction
SNF: Similarity Network Fusion
SNV: single-nucleotide variant
SV: structural variant
TF: Transcription Factor
TieDIE: Tied Diffusion Through Interacting Events
