# Supplementary material for: Computational methods for cancer driver discovery: A survey
Source: Theranostics. 2021 Mar 20;11(11):5553–68. doi: 10.7150/thno.52670 (PMC8039954; doi:10.7150/thno.52670)
Supplement: Supplementary file 1 — Supplementary figures and tables. [file thnov11p5553s1.pdf]

Supplement to "Computational methods for cancer driver discovery: A survey"

Vu Viet Hoang Pham[1], Lin Liu[1], Cameron Bracken[2,3], Gregory Goodall[2,3], Jiuyong Li[1], Thuc Duy Le[1*]

1 UniSA STEM, University of South Australia, Mawson Lakes, SA 5095, AU
2 Centre for Cancer Biology, SA Pathology, Adelaide, SA 5000, AU
3 Department of Medicine, The University of Adelaide, Adelaide, SA 5005, AU

* Thuc.Le@unisa.edu.au

1 Network-based methods

Network-based methods use gene networks to assess the role of genes then combine with the mutation information to predict cancer driver genes. The general idea of the network-based methods is illustrated in Figure 1.

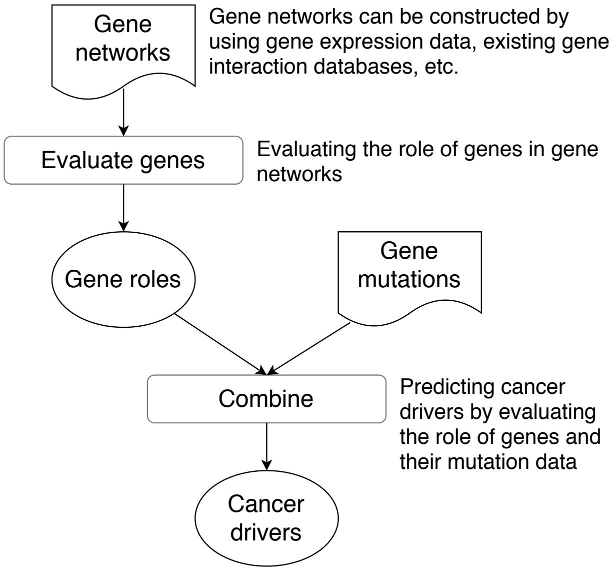

Fig 1. Network-based methods. Network-based methods evaluate the role of genes in gene regulatory networks by using different techniques and combine with the mutations of genes to predict cancer drivers.

2 Resources for cancer driver research

There are two types of resources for developing computational methods for cancer driver discovery, including the resources for method development, e.g. gene expression data, network data, mutation data, etc; and the resources for gene annotations, e.g. a database with partial ground truth for evaluating or assessing the findings of a computational method. The resources are summarised in Table 1.

Table 1. Summary of resources for cancer driver research.

Resource	Website	Description and reference
Resource for method development		
TCGA	https://www.cancer.gov/about-nci/organization/ccg/research/structural-genomics/tcga	Profiles human tumours to discover molecular aberrations in DNA, XRNA, protein, and epigenetic levels [1]
ICGC	https://icgc.org/	A data portal of cancer gemomics of 50 cancer types [2]
cBioPortal	http://www.cbioportal.org/	A web interface for accessing cancer genomic data and analysing the data [3]
Cancer3D	http://cancer3d.org/search	Contains mutations of more than 14,700 proteins and they are mapped to proteins of the Protein Data Bank [4] (over 24,300 proteins in the bank) [5]
CCLE	https://portals.broadinstitute.org/ccle	Includes SNVs, CNAs, and gene expression [6]
COSMIC	https://cancer.sanger.ac.uk/cosmic	Contains cancer mutations, including manually curated expert data and data from sequencing projects [7]
Resource for gene annotations		
CGC	https://cancer.sanger.ac.uk/census	Provides driver genes which are manually curated or predicted by multiple methods [8]
AGCOH	http://atlasgeneticsoncology.org/	Contains about 1,500 cancer genes merged from numerous collaborative projects [9]
NCG	http://ncg.kcl.ac.uk/	Comprises more than 500 known cancer genes and over 1,000 candidate cancer genes [10]
DGIdb	http://www.dgidb.org/	Includes cancer drivers and drug-gene interactions [11]
OncomiR	http://www.oncomir.org/	A web interface for investigating miRNA dysregulation [12]

About resource for method development, several databases have been developed from cancer sequencing projects and they provide rich data used in cancer driver identification methods. TCGA [1] is a significant project in this area. The TCGA project profiles and analyses human tumours to uncover molecular aberrations in DNA, XRNA, protein, and epigenetic levels [1]. TCGA data can be accessed through the Genomic Data Commons (GDC) data portal [13]. ICGC data portal is also a resource for cancer genomics data and it contains the data of genomic abnormalities of 50 cancer types [2]. Another data portal for cancer genomics is cBioPortal [3], which provides a web interface for accessing cancer genomic datasets, as well as for analysing and visualising the data online.

There are also some other resources which can be used for cancer driver discovery such as the Cancer3D [5], the Cancer Cell Line Encyclopedia (CCLE) [6], and the COSMIC database [7]. Cancer3D is a database which focuses on the influence of mutations on the structure of proteins and it provides the information for users to analyse distribution patterns of mutations and their relationship with changes in drug activity [5]. It contains mutations of more than 14,700 proteins, which are mapped to over 24,300 proteins in the Protein Data Bank [4]. The CCLE includes SNVs, CNAs, and gene expression [6]. The COSMIC database is a large and comprehensive source for investigating the mutational impact in cancer. It contains records of cancer mutations including both manually curated expert data and data from sequencing projects like TCGA or ICGC [7, 14]. It has more than two million coding point mutations and over six million non-coding mutations [7].

As about the resources for gene annotations, currently several databases such as the CGC [8] (in COSMIC) can be used. The CGC contains driver genes which are manually curated or predicted by multiple methods. Beside the CGC, several other sources are available for gene annotations. The Atlas of Genetics and Cytogenetics in Oncology and Haematology (AGCOH) is another source for this purpose [9]. It comprises around 1,500 cancer genes which are merged results from numerous collaborative projects [9]. The Network of Cancer Genes (NCG) is an online database of cancer genes with over 500 known cancer genes and more than 1,000 candidate cancer genes [10]. Known cancer genes are genes which have already been confirmed through experiments while candidate cancer genes are those using statistical methods. One more database about disease genes is the Drug-Gene Interaction database (DGIdb) [11]. It contains not only cancer drivers but also the information about drugs and drug-gene interactions [11].

At the present, while coding drivers are established in cancer research, non-coding drivers are not. In [12], the authors have recently introduced OncomiR, which is a resource for investigating miRNA dysregulation in cancer through a web interface. It does statistical analyses based on RNA-seq, miRNA-seq, and clinical information from TCGA to discover miRNAs which are related to cancer progression. Although this database may not be used as a ground truth to validate miRNA cancer drivers, it can be used as a channel to explore miRNA dysregulation in detecting miRNA cancer drivers. To validate non-coding cancer drivers now, it is required to examine the literature manually [15, 16].

3 Driver genes predicted by different methods

There are 63 breast cancer drivers predicted by at least by two of the five methods (DriverML, ActiveDriver, DriverNet, MutSigCV, and OncodriveFM). The details of these 63 drivers are presented in Table 2. We also evaluate the mutation frequency of these driver genes by using the breast mutation data downloaded from TCGA. We only select somatic mutations which are functional based on the variant classification of mutations, such as splice_site, in_frame_del, and frame_shift_del. To validate these driver genes, we use the CGC from the COSMIC database [7] as a gold standard. The CGC is a commonly used cancer gene database for validating cancer drivers predicted by computational methods in cancer research. It can be seen from the table that most of the predicted driver genes are mutated genes. Especially, the 11 driver genes which are predicted by at least three methods have a high mutation frequency. In addition, all these 11 driver genes are in the CGC. Although computational methods may never completely replace wet laboratory experiments in biological research, the novel drivers predicted by these methods can be used as candidates for further wet laboratory experiments to confirm their roles in cancer development. Some potential breast cancer drivers discovered by these methods include RBMX, NCOA3, and ZFP36L1. There is evidence showing that a positive correlation exists between the expression of RBMX and the proapoptotic Bax gene in breast cancer patients [17]. NCOA3 is also known to regulate PERK-eIF2α-ATF4 signalling [18] and activates estrogen receptor α-mediated transactivation of PLAC1 in breast cancer [19]. ZFP36L1 has been show to suppress HIF1α and Cyclin D1 in breast cancer [20].

Table 2. The list of breast cancer driver genes predicted by different methods.

No.	Driver	Predicted by methods	Number of methods	Mutation frequency	In CGC?
1	TP53	DriverML,ActiveDriver,MutSigCV, OncodriveFM,DriverNet	5	328	✓
2	CDH1	DriverML,MutSigCV,OncodriveFM, DriverNet	4	119	✓
3	PIK3CA	DriverML,MutSigCV,OncodriveFM, DriverNet	4	381	✓
4	GATA3	DriverML,ActiveDriver,MutSigCV	3	104	✓
5	NCOR1	DriverML,ActiveDriver,MutSigCV	3	45	✓
6	PTEN	DriverML,MutSigCV,OncodriveFM	3	39	✓
7	ARID1A	DriverML,ActiveDriver,MutSigCV	3	30	✓
8	FOXA1	DriverML,MutSigCV,OncodriveFM	3	25	✓
9	PIK3R1	DriverML,ActiveDriver,MutSigCV	3	18	✓
10	CTCF	DriverML,ActiveDriver,MutSigCV	3	17	✓
11	ERBB2	ActiveDriver,MutSigCV,OncodriveFM	3	23	✓
12	AOAH	DriverML,MutSigCV	2	1	
13	MAP3K1	DriverML,MutSigCV	2	103	✓
14	RBMX	DriverML,MutSigCV	2	14	
15	RUNX1	DriverML,MutSigCV	2	34	✓
16	NCOR2	DriverML,MutSigCV	2	8	✓
17	BAX	DriverML,MutSigCV	2	11	✓
18	SPEN	DriverML,ActiveDriver	2	47	✓
19	NCOA3	DriverML,MutSigCV	2	9	
20	RBM5	DriverML,MutSigCV	2	5	
21	CBFB	DriverML,MutSigCV	2	25	✓

22	MUC12	DriverML,MutSigCV	2	84	
23	ZFP36L1	DriverML,MutSigCV	2	9	
24	HRNR	DriverML,ActiveDriver	2	41	
25	CDKN1B	DriverML,MutSigCV	2	12	✓
26	USP36	DriverML,MutSigCV	2	8	
27	RPGR	DriverML,MutSigCV	2	24	
28	ASB10	DriverML,MutSigCV	2	9	
29	ACTL6B	DriverML,MutSigCV	2	10	
30	NR1H2	DriverML,MutSigCV	2	8	
31	MEF2A	DriverML,MutSigCV	2	6	
32	ZNF384	DriverML,MutSigCV	2		✓
33	ATN1	DriverML,MutSigCV	2	15	
34	THEM5	DriverML,MutSigCV	2	11	
35	AQP7	DriverML,MutSigCV	2	2	
36	C1QTNF5	DriverML,MutSigCV	2	11	
37	FNDC4	DriverML,MutSigCV	2	6	
38	MAPRE3	DriverML,MutSigCV	2	9	
39	SH3PXD2A	DriverML,MutSigCV	2	13	
40	CCDC144NL	DriverML,MutSigCV	2		
41	TAF1B	DriverML,MutSigCV	2	9	
42	FAT3	DriverML,OncodriveFM	2	39	✓
43	C9orf43	DriverML,MutSigCV	2	12	
44	MAP2K4	DriverML,MutSigCV	2	33	✓
45	EPDR1	DriverML,MutSigCV	2	5	
46	KCNN2	DriverML,ActiveDriver	2	5	
47	BCL6B	DriverML,MutSigCV	2	3	
48	GPS2	DriverML,MutSigCV	2	11	
49	U2AF2	DriverML,MutSigCV	2	4	
50	SETDB1	DriverML,ActiveDriver	2	16	
51	ZFP36L2	DriverML,MutSigCV	2	7	
52	CDSN	DriverML,MutSigCV	2		
53	LCT	DriverML,ActiveDriver	2	17	
54	SLC25A5	DriverML,MutSigCV	2	2	
55	VEZF1	DriverML,MutSigCV	2	7	
56	HERC1	DriverML,ActiveDriver	2	29	
57	CDC27	ActiveDriver,OncodriveFM	2	8	
58	RELN	ActiveDriver,OncodriveFM	2	28	
59	AKT1	MutSigCV,OncodriveFM	2	2	✓
60	ZNF302	MutSigCV,OncodriveFM	2		
61	SF3B1	MutSigCV,OncodriveFM	2	18	✓
62	TPRX1	MutSigCV,OncodriveFM	2	6	
63	GPR32	MutSigCV,OncodriveFM	2	5	

References

1. Institute NHGR. The cancer genome atlas. 2018;.

2. Zhang J, Baran J, Cros A, Guberman JM, Haider S, Hsu J, et al. International cancer genome consortium data portal: a one-stop shop for cancer genomics data. Database. 2011;2011:bar026–bar026.

3. Gao J, Aksoy BA, Dogrusoz U, Dresdner G, Gross B, Sumer SO, et al. Integrative analysis of complex cancer genomics and clinical profiles using the cBioPortal. Sci Signal. 2013;6(269):pl1.

4. Rose PW, Bi C, Bluhm WF, Christie CH, Dimitropoulos D, Dutta S, et al. The RCSB protein data bank: new resources for research and education. Nucleic Acids Res. 2013;41(Database issue):D475–D482.

5. Porta-Pardo E, Hrabe T, Godzik A. Cancer3D: understanding cancer mutations through protein structures. Nucleic Acids Res. 2015;43(Database issue):D968–D973.

6. Barretina J, Caponigro G, Stransky N, Venkatesan K, Margolin AA, Kim S, et al. The cancer cell line encyclopedia enables predictive modeling of anticancer drug sensitivity. Nature. 2012;483(7391):603–607.

7. Forbes SA, Beare D, Gunasekaran P, Leung K, Bindal N, Boutselakis H, et al. COSMIC: exploring the world's knowledge of somatic mutations in human cancer. Nucleic Acids Res. 2015;43(Database issue):D805–D811.

8. Futreal PA, Coin L, Marshall M, Down T, Hubbard T, Wooster R, et al. A cencus of human cancer genes. Nat Rev Cancer. 2004;4(3):177–183.

9. Huret JL, Minor SL, Dorkeld F, Dessen P, Bernheim A. Atlas of genetics and cytogenetics in oncology and haematology, an interactive database. Nucleic Acids Res. 2000;28(1):349–351.

10. An O, Dall'Olio GM, Mourikis TP, Ciccarelli FD. NCG 5.0: updates of a manually curated repository of cancer genes and associated properties from cancer mutational screenings. Nucleic Acids Res. 2016;44(Database issue):D992–D999.

11. Griffith M, Griffith OL, Coffman AC, Weible JV, McMichael JF, Spies NC, et al. DGIdb: mining the druggable genome. Nat Methods. 2013;10:1209.

12. Wong NW, Chen Y, Chen S, Wang X. OncomiR: an online resource for exploring pan-cancer microRNA dysregulation. Bioinformatics. 2018;34(4):713–715.

13. Grossman RL, Heath AP, Ferretti V, Varmus HE, Lowy DR, Kibbe WA, et al. Toward a shared vision for cancer genomic data. N Engl J Med. 2016;375(12):1109–1112.

14. Forbes SA, Bindal N, Bamford S, Cole C, Kok CY, Beare D, et al. COSMIC: mining complete cancer genomes in the catalogue of somatic mutations in cancer. Nucleic Acids Res. 2011;39(Database issue):D945–D950.

15. Cuykendall TN, Rubin MA, Khurana E. Non-coding genetic variation in cancer. Curr Opin Syst Biol. 2017;1:9–15.

16. Poulos RC, Sloane MA, Hesson LB, Wong JW. The search for cis-regulatory driver mutations in cancer genomes. Oncotarget. 2015;6(32):32509–32525.

17. Martínez-Arribas F, Agudo D, Pollán M, Gómez-Esquer F, Díaz-Gil G, Lucas R, et al. Positive correlation between the expression of X-chromosome RBM genes (RBMX, RBM3, RBM10) and the proapoptotic Bax gene in human breast cancer. J Cell Biochem. 2006;97(6):1275–82.

18. Gupta A, Hossain MM, Miller N, Kerin M, Callagy G, Gupta S. NCOA3 coactivator is a transcriptional target of XBP1 and regulates PERK–eIF2α–ATF4 signalling in breast cancer. Oncogene. 2016;35(45):5860–5871.

19. Wagner M, Koslowski M, Paret C, Schmidt M, Özlem Türeci, Sahin U. NCOA3 is a selective co-activator of estrogen receptor α-mediated transactivation of PLAC1 in MCF-7 breast cancer cells. BMC Cancer. 2013;13(1):570.

20. Loh XY, Ding LW, Koeffler HP. Abstract 4494: Tumor suppressive role of ZFP36L1 by suppressing HIF1α and Cyclin D1 in bladder and breast cancer. Cancer Research. 2017;77(13 Supplement):4494–4494.
